# Surveillance Programme of IN-patients and Epidemiology (SPINE): Implementation of an Electronic Data Collection Tool within a Large Hospital in Malawi

**DOI:** 10.1371/journal.pmed.1001400

**Published:** 2013-03-12

**Authors:** Miguel A. SanJoaquin, Theresa J. Allain, Malcolm E. Molyneux, Laura Benjamin, Dean B. Everett, Oliver Gadabu, Camilla Rothe, Patrick Nguipdop, Moses Chilombe, Lawrence Kazembe, Servace Sakala, Andrew Gonani, Robert S. Heyderman

**Affiliations:** 1 Malawi-Liverpool-Wellcome Trust Clinical Research Programme, University of Malawi College of Medicine, Blantyre, Malawi; 2 Liverpool School of Tropical Medicine, Liverpool, United Kingdom; 3 Department of Medicine, University of Malawi College of Medicine, Queen Elizabeth Central Hospital, Blantyre, Malawi; 4 Baobab Health, Lilongwe, Malawi; 5 Chancellor College, Zomba, Malawi; 6 Queen Elizabeth Central Hospital, Blantyre, Malawi

## Abstract

Miguel Sanjoaquin and colleagues describe their experience of setting up an electronic patient records system in a large referral hospital in Blantyre, Malawi.

**Summary Points**

- Disease burden in developing countries is difficult to quantify—data are sparse and their quality is difficult to assess.
- Health care planning and the monitoring and evaluation of interventions are hindered by a lack of systematically collected data.
- An electronic surveillance system was set up to gather morbidity and mortality data from adult patients in a large referral hospital of Malawi (Surveillance Programme of IN-patients and Epidemiology [SPINE]), serving as a model for other potential sentinel implementation sites.
- SPINE collects individual-level demographic and clinical data from thousands of admissions, thereby informing patient care, policy planning, and research.
- We discuss the strengths and weaknesses of establishing these types of systems and the challenges that were overcome to implement SPINE.

## The Challenge

In low-income countries, a prominent obstacle to the effective planning and delivery of health care is the accurate assessment of the disease burden. Commonly used paper-based systems for recording hospital admissions in countries such as Malawi are cumbersome, of uncertain reliability, and do not lend themselves to regular analysis or feedback to clinical teams or health care planners. The Ministry of Health in Malawi and its partners have been at the forefront of the roll-out of a number of major interventions to reduce the burden of HIV [1], and tuberculosis and malaria [2]. The monitoring of progress and the evaluation of success of such programmes is of critical importance but such parallel activities are costly, tend to interfere with the effects of the interventions, and may distort disease estimates [3]. Disease surveillance at sentinel hospital facilities has the potential to circumvent these problems [4]–[7].

Almost a decade ago a touch screen electronic data system that centralizes outpatient clinical records of patients receiving antiretroviral drugs (ARVs) was introduced in a large referral hospital in southern Malawi, which was subsequently rolled out across the country [8]. We set out to expand the electronic data collection system beyond the HIV service with the aim of improving care for patients and improving disease surveillance. Now we show how this electronic data collection system can be extended to successfully provide ready access to the clinical records of patients admitted to the adult medical wards where the impact of the HIV epidemic is greatest (approximately 70% of patients are HIV-infected), and where care is also provided for a large volume of patients with a wide range of communicable and non-communicable diseases. The undertaking resulted in a long process requiring considerable engagement and sensitization but was ultimately worth the effort.

## The Project

### The Surveillance Programme of IN-patients and Epidemiology

The Surveillance Programme of IN-patients and Epidemiology (SPINE), an unrelated system to the one set up in the UK, records information through touch-screen terminals located in the hospital adult internal medical wards. The electronic system is a £200,000 investment that took 8 months to develop and 3 months to implement, including sensitization and training. Coding is based on open source software named Ruby (http://www.ruby-lang.org/en/) and therefore allows the sharing of software packages created in different hospitals and from different initiatives at no added cost. The screens are generated from standard HTML web forms using the touch-screen Toolkit. The electronic medical record (EMR) is a password-protected web-based application developed using completely open source platforms. SPINE is connected to the ARV clinic system, enabling patients registered in either system to be recognized by both. When a patient is discharged from hospital a member of the clinical team looking after the patient records the HIV status, ARV use, primary diagnosis, and up to two secondary diagnoses. For this purpose the electronic system provides a series of user-friendly screens allowing diagnoses to be chosen from a comprehensive list modified from the International Classification of Diseases (ICD-10). The clinician can also formulate a prescription, which is linked to the diagnosis made. The system provides access to a limited past medical history, including previous diagnoses and treatment received. On discharge, a summary of the patient's visit, including the discharge medication is printed on an adhesive label and place in the patient's health passport. This avoids the need for hand written discharge summaries and transcription errors. Terminals are password protected to ensure patient's privacy. When health care workers have to log in, the user initiates a session by scanning the barcoded label on the patient's health passport or hospital notes. The health passport is a document that the vast majority of patients (>90%) carry to be seen at hospital or health centers. If not, they can buy one on entry into the health facility at a token price. The terminals are used for the registration of patients, from whom basic demographic information is collected and if a new patient, a unique barcode identifier is generated. Duplicate registrations are largely avoided through a well-validated cross-checking algorithm that uses critical personal variables such as name, age, sex, and location. The electronic system co-exists with the other standard paper-based data collection activities needed for lab, clinical, and hospital functioning. These serve as data back-up and information is sometimes retrieved from these sources when a backlog happens, due to, for example, prolonged power outages. The long-term plan is to discontinue some of these paper-based data collection activities once SPINE is thought to be stable.

### A Tool for Patient Care

The most immediate benefit for the patient is the easy-to-read print out that contains information on diagnosis, laboratory tests, and prescription made. The prescription is presented in a simple way that patients can understand. Indirect benefits include a facility for clinicians to retrieve past medical history. As many adult in-patients on the medical wards are HIV infected and re-admissions are a common phenomenon, having this information, even if other records are missing, has the potential to improve management. SPINE is thought to be having a positive impact on patient care by prompting clinicians to remember HIV testing, facilitating clear management plans, and improving quality of prescribing by giving clear printed prescriptions and correctly spelt drug names with appropriate doses. It is a suitable tool for auditing, monitoring diagnosis, and prescription patterns. SPINE can be used for mapping outbreaks. Deviation from good practice is discussed in review sessions with the medical staff. Quantifying impact on patient care is an important target for the implementers of SPINE. The system is already reinforcing existing policies (e.g., HIV testing). Performance indicators such as re-admission and mortality rates are being monitored and as more data are accumulated, we will be able to determine whether this system, amid multiple other interventions and changes in medical practice, is having a positive effect.

### Initial Data Generated by SPINE

SPINE is based within the Department of Medicine (adult internal medicine admissions) of Queen Elizabeth Central Hospital (QECH), one of the largest hospitals of Malawi. Previously paper-based recording of hospital visits and admissions through health management information systems (HMIS) at QECH were the norm.

SPINE has been operational at QECH since late 2009. It has had a positive uptake from the clinical staff and, at present, most doctors and nurses are conversant with its use and utilize SPINE to make discharges, despite the initially low computer literacy in some cases. A summary of the basic descriptors for the adults admitted during the period January 2010 to July 2011 is provided in Table 1. Data collection was temporarily interrupted after July 2011 to allow for some major structural modifications in the hospital buildings. HIV status was available for 79% of all recorded admissions and three in four were HIV positive. HIV positive patients spent more time admitted and were more likely to die compared to HIV negative patients. In-hospital mortality was substantial (15% of all admissions).

**Table 1:** Summary statistics.

Characteristic	*n* (%)
**Total admissions**	7,103
in 2010	4,699
in 2011^a^	2,404
*n* patients diagnosed with 2 conditions	1,201
**Age** (median)	37
**Sex ratio**	1∶1
**HIV**	
Reactive^b^	1,450 (26)
Non-reactive^b^	4,223 (74)
Unknown	1,430 (21)
**Days admitted** (median)	5
**Outcome** c	
Alive	6,031 (85)
Dead	1,036 (15)

a Enrolment interrupted after July 2011.

b Percentage excludes unknown.

c 36 patients absconded.

### Morbidity, Mortality, and Case-Fatality Rate

The total number of patients admitted by disease condition, the fraction of them that died in hospital, and prevalence of HIV infection is shown in Table 2. Infectious diseases were the top five most frequent causes of admission (pneumonia, tuberculosis, sepsis, meningitis, and malaria), accounted for more than half of all admissions, and were normally associated with HIV infection. However, the admission pattern was slightly different among HIV negative patients where heart disease and stroke were the third and fourth most prevalent causes of admission (unpublished data). Meningitis was the commonest cause of death and also the disease associated with the greatest chance of dying.

**Table 2:** Morbidity, mortality, and HIV prevalence.

Disease	*n*	Percent Died	Percent HIV Infected
Pneumonia	1,149	10	84
Tuberculosis	1,003	17	88
Other	973	9	62
Sepsis	637	12	82
Meningitis	531	36	86
Malaria	502	5	63
Upper respiratory infection	301	1	74
Non-communicable heart disease	277	16	36
Acute gastroenteritis	247	15	79
Stroke	236	21	43
Anaemia	224	19	71
Neoplasia	222	23	61
Chronic lung disease	212	1	46
Hypertension	100	5	45
Cirrhosis/hepatitis	93	31	48
Renal disease	91	18	63
Urinary tract infection	79	3	63
Diabetes	78	3	29
Epilepsy	76	3	27
Peripheral neuropathy	72	8	83

### Seasonal Fluctuation

**Figure 1:**
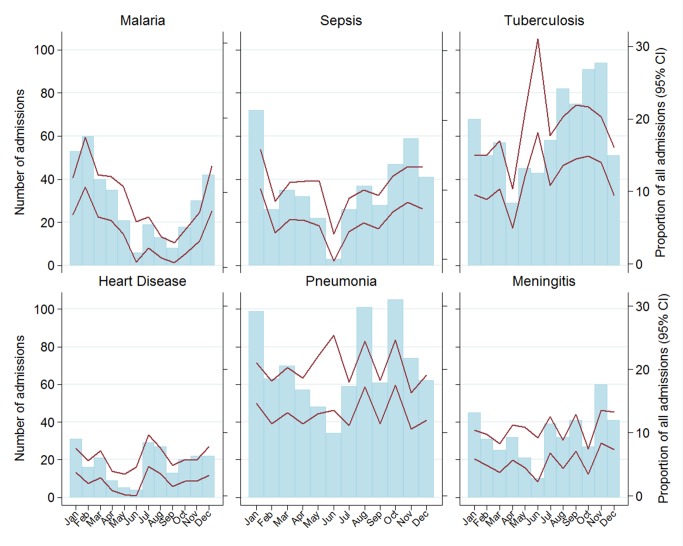
Number of admissions (bars) and its proportion of all admissions (lines, with 95% confidence intervals).

The pattern of disease and the frequency of admissions were markedly influenced by season, highlighting that health planning and clinical assessment will need to take these into account. The Malawi rainy season is typically between December and April, and the cold season between June and August. There was a peak in disease-specific admissions between January and March (Figure 1). Malaria and sepsis followed a similar pattern of admission, as compared to the annual average, in the months of January and February. The maximum number of malaria cases was in March, after the peak of the rainy season. Heart disease and stroke peaked in September and March. Neither pneumonia nor meningitis showed marked seasonal increases, which may be because in adults both are dominated by HIV co-infection [2].

## Challenges and Lessons Learnt

Although similar systems are being implemented elsewhere, experience with creating electronic patient record systems in resource-poor settings is still scarce [2]. Few of these systems have been formally evaluated, even in resource-rich countries where electronic medical record (EMR) development is challenging. We have found that in everyday use, such a system can be successfully implemented to provide vital morbidity and mortality data and makes individual patient information accessible at the point of care. Training and sensitization are essential ingredients of success. The fundamental area of training involves mainly the clinicians and how to use the system; this consists of a one-off session followed by continued support at the ward. Regular sensitization with medical staff, where the sometimes not so obvious long-term benefits of the system are explained, proved to be important and bolstered the utilization rate. Prolonged power outages and system failures (both hardware and software), however infrequent, continue to be a significant concern and have the potential to undermine confidence in the system. Taking into consideration the electrical power interruptions in Malawi, low power solutions have been adopted so that the system continues to operate in times of power failures. These solutions consist of a set of accumulators and invertors that are charged while power is on and can supply electricity for up to 72 hours when power failures occur, which was sufficient to prevent a negative impact from over 90% of all power outages. Patient confidentiality is ensured through password protection and controlled access to the data. Patients themselves are very accepting of the electronic system and indeed show considerable interest.

### The Value of Using Hospitals for Surveillance

One of the main obstacles in monitoring of the success of international public health targets, including the Millennium Development Goals (MDGs) [9], has been the lack of accurate monitoring and evaluation (M&E) of disease burdens. Reliable and comparable information about the main causes of disease in populations and how these causes are changing, is a crucial input for debates about priorities in the health sector [10],[11]. Sentinel hospital surveillance has the advantage that data can be collected at a larger scale and for less financial investment compared to community surveys; further it covers larger geographical areas and enables a more well-defined diagnosis to be made. Electronic systems such as SPINE make these data easily accessible and relatively cheap to record, providing nationally and internationally comparable information on severe illness indicators and their changeover time. In addition, these systems have the potential to improve the quality of health care planning and delivery.

### Strength and Limitations of SPINE

Currently, clinicians lead data entry into the system. This has the advantage of being a more sustainable approach and promotes a feeling of ownership. Involvement with data entry encourages clinicians to ensure the accuracy of discharge diagnoses and to make use of the medical information. However, in the context of a hospital that sees approximately 250,000 patients a year and admits approximately 25,000 adults, using an electronic system places an extra burden on clinicians and this was the main roadblock in the implementation phase. On average, it is estimated that each discharge takes 2–3 minutes for a clinician conversant with the system. This means that, at least for an interim period, one additional clinical officer and four nurses had to be employed to assist with training, technical support, and some of the discharges. The initial reluctance to use the system diminished over time once SPINE was populated with enough previous admissions for it to be useful for clinicians. Lack of accuracy in the diagnosis and missed discharges are other factors that compromise the integrity and validity of the data. At the time of writing this article, SPINE was capturing over 80% of all discharges. Regarding the quality of data, a number of independent unpublished research studies conducted concurrently have provided an indication of the quality of diagnosis. For example, a study looking into meningitis case-fatality rate showed that 50% of all patients diagnosed with meningitis died, which is compatible with the 36% seen by SPINE. The burden of severe malaria admission was 7% of all admissions as measured by an enhanced surveillance activity and 8% when using SPINE. These electronic tools have also required readily available IT/programming expertise to develop and maintain these relatively sophisticated systems. Failures in the system have occurred and these have the potential to reduce motivation, resulting in additional patients not being discharged through the system.

### Moving Forward

Our experience with SPINE demonstrates that such a system can be successfully used for large-scale surveillance, facilitation of in-patient management, and as a platform for clinical research. SPINE can also be used by the hospital administration for monitoring clinical activity, as an audit tool, and for drug forecasting. Our next steps will be to connect SPINE with the laboratory services. We are developing a robust data reporting system for health authorities and hospital management to have updated information and generating a real-life outbreak alert system using geographical information technology. Other health facilities where the capacity for maintenance exists could also benefit from such systems.

## Abbreviations

ARV: antiretroviral
QECH: Queen Elizabeth Central Hospital
SPINE: Surveillance Programme of IN-patients and Epidemiology
